# Population-based incidence and comparative demographics of community-associated and healthcare-associated *Escherichia coli* bloodstream infection in Auckland, New Zealand, 2005 – 2011

**DOI:** 10.1186/1471-2334-13-385

**Published:** 2013-08-21

**Authors:** Deborah A Williamson, Alwin Lim, Siouxsie Wiles, Sally A Roberts, Joshua T Freeman

**Affiliations:** 1 Faculty of Medical and Health Sciences, University of Auckland, Auckland, New Zealand; 2 Department of Clinical Microbiology, Auckland District Health Board, Park Road, Auckland, Grafton, New Zealand; 3 Institute of Environmental Science and Research, Wellington, New Zealand

**Keywords:** *Escherichia coli*, Bacteremia, Antimicrobial resistance

## Abstract

**Background:**

*Escherichia coli* is a major human pathogen, both in community and healthcare settings. To date however, relatively few studies have defined the population burden of *E. coli* bloodstream infections. Such information is important in informing strategies around treatment and prevention of these serious infections. Against this background, we performed a retrospective, population-based observational study of all cases of *E. coli* bacteremia in patients presenting to our hospital between January 2005 and December 2011.

**Methods:**

Auckland District Health Board is a tertiary-level, university-affiliated institution serving a population of approximately 500,000, within a larger metropolitan population of 1.4 million. We identified all patients with an episode of bloodstream infection due to *E. coli* over the study period. A unique episode was defined as the first positive *E. coli* blood culture taken from the same patient within a thirty-day period. Standard definitions were used to classify episodes into community- or healthcare-associated *E. coli* bacteremia. Demographic information was obtained for all patients, including: age; gender; ethnicity; length of stay (days); requirement for intensive care admission and all-cause, in-patient mortality.

**Results:**

A total of 1507 patients had a unique episode of *E. coli* bacteremia over the study period. The overall average annual incidence of *E. coli* bacteremia was 52 per 100,000 population, and was highest in the under one year and over 65-year age groups. When stratified by ethnicity, rates were highest in Pacific Peoples and Māori (83 and 62 per 100,000 population respectively). The incidence of community-onset *E. coli* bacteremia increased significantly over the study period. The overall in-hospital mortality rate was 9% (135/1507), and was significantly higher in patients who had a hospital-onset *E. coli* bacteremia.

**Conclusions:**

Our work provides valuable baseline data on the incidence of *E. coli* bacteremia in our locale. The incidence was higher that that described from other developed countries, with significant demographic variation, most notably in ethnic-specific incidence rates. Future work should assess the possible reasons for this disparity.

## Background

*Escherichia coli* is a major human pathogen, and infections caused by *E. coli* result in significant morbidity and mortality [1]. Although most commonly associated with uncomplicated urinary tract infections, extraintestinal strains of *E. coli* can also cause a wide variety of serious infections, including meningitis, pneumonia and bacteremia [1]. Previous studies have consistently ranked *E. coli* as the most common cause of community-onset bacteremia, and a major causative pathogen in nosocomial bacteremia [2-6]. Over the past decade, several countries have described an increase in the incidence of *E. coli* bloodstream infections (EC-BSI) [7,8]. In the United Kingdom, this phenomenon has resulted in the introduction of mandatory surveillance of EC-BSI in order to investigate the possible factors underlying the increase in these serious infections [8,9].

Over the past two decades, treatment of EC-BSI has become increasingly complicated by the emergence of antimicrobial-resistant *E. coli* strains. In particular, acquired resistance due to genes encoding extended-spectrum beta-lactamases (ESBLs) and carbapenemases poses a significant therapeutic challenge, and bloodstream infections with these resistant organisms have been associated with adverse clinical and economic consequences [10,11].

However, despite the significant morbidity and mortality associated with EC-BSI, relatively few studies have attempted to define the population burden of these important infections [5,12]. In addition, although sociodemographic disparities have been described for other major causes of bloodstream infections such as *Staphylococcus aureus*[13] and *Streptococcus pneumoniae*[14], little data exists on the potential demographic variation in episodes of EC-BSI. Such population-based epidemiological information is important in informing strategies around treatment and prevention of these serious infections.

Accordingly, we sought to assess the incidence, antimicrobial-resistance trends and outcomes of EC-BSI in patients presenting to our hospital between January 2005 and December 2011, with particular regard to any variation in demographic characteristics. In addition, we sought to describe any demographic variation in the incidence of community-associated (CA) vs. healthcare-associated (HCA) EC-BSI in our locale.

## Methods

### Setting and study design

Auckland District Health Board (ADHB) is a tertiary-level, university-affiliated institution which exclusively serves a population of approximately 500,000, within a larger metropolitan region of 1.4 million. Auckland is the largest city in New Zealand, and has an ethnically diverse population, consisting of the following major population groups: European (52%); Asian (29%), Pacific Island peoples (11%); Māori (indigenous New Zealander, 8%) and other ethnicities (2%) [15].

We performed a retrospective cross-sectional study of all patients at ADHB with EC-BSI between January 2005 and December 2011. Cases of EC-BSI were identified from the laboratory database in the Department of Clinical Microbiology, Auckland City Hospital, New Zealand. A unique episode was defined as the first positive *E. coli* blood culture taken from the same patient within a thirty-day period.

### Data collection and definitions

Using information extracted from the hospital administrative database, the following demographic information was obtained for each patient: age, gender, ethnicity, domicile, and number of previous hospitalizations in the preceding year. In addition, we also extracted all hospital discharge diagnoses related to each episode of EC-BSI, coded using the *International Classification of Diseases, Tenth Edition, Clinical Modification* (*ICD-10-CM*) codes [16].

In keeping with previous methodology [17], cases were described as community-associated EC-BSI (CA EC-BSI) if *E. coli* was isolated from the bloodstream of a patient within 48 hours of hospital admission who: (i) had no history of hospitalization or surgery in the preceding calendar year, and (ii) did not reside in a long-term care facility (LTCF), and (iii) did not have any prior or current *ICD-10-CM* discharge diagnoses relating to hemodialysis. Conversely, cases were described as healthcare-associated EC-BSI (HCA EC-BSI) if one or more of these risk factors were documented. Healthcare-associated cases were further described as hospital-onset (HCA-HO) or community-onset (HCA-CO) depending on whether the specimen was taken > 48 hours or ≤ 48 hours respectively, following hospital admission.

Population denominator information was obtained from the 2006 New Zealand census, and from projected population data for the Auckland region [15]. For analysis, ethnicity was grouped into four major ethnic groupings: European, Māori, Pacific Peoples and Asian/other ethnicities.

The following clinical outcome measures were obtained for all patients: length of hospital stay (days), requirement for intensive care unit admission, and all-cause in-patient hospital mortality. For all patients who had hospital-onset EC-BSI, the time (in days) from admission to first positive blood culture was calculated.

### Microbiological analysis

All *E. coli* isolates were identified using the RapID One system (Remel Diagnostics). Susceptibility testing was performed using agar dilution in keeping with Clinical and Laboratory Standards (CLSI) recommendations for the following antimicrobials: amoxicillin, amoxicillin-clavulanate, ticarcillin-clavulanate, cephalothin, cefuroxime, ceftazidime, ceftriaxone, gentamicin, amikacin, trimethoprim-sulphamethoxazole, ciprofloxacin and meropenem [18]. Isolates testing as intermediate were classified as resistant. ESBL and / or carbapenemase production was detected according to CLSI recommendations [18]. An isolate was classified as multidrug-resistant (MDR) if it displayed resistance to at least one representative of ≥ 3 classes of antimicrobial agents, as previously described [19].

### Statistical analysis

Categorical variables were compared using either the χ^2^ or Fisher’s exact test as appropriate. Non-parametric data were expressed as median values with interquartile rages (IQR), and were compared using the Mann–Whitney *U* test or Kruskal-Wallis analysis of variance (ANOVA) test. Incidence rates were calculated per 100,000 population, and were stratified according to age, gender and ethnicity. A Poisson log-linear regression model was used to assess trends in incidence rates over time using log population denominator data as the offset variable. All statistical analysis was performed using GraphPad Prism (Version 5.02) or STATA (Version 11) and a two-tailed *P* value of < 0.05 was considered significant.

### Ethics

The Auckland District Health Board, New Zealand, granted institutional approval for this study.

## Results

### Patients and incidence rates of E. coli bacteremia

**Figure 1:**
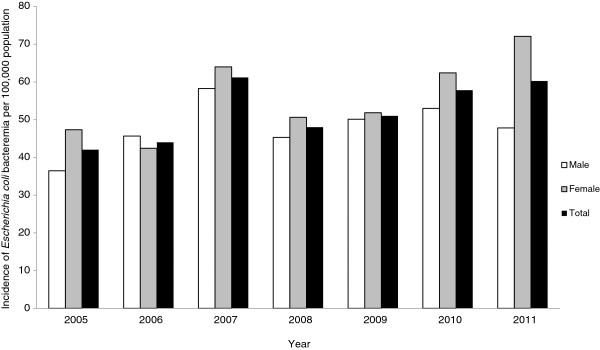
**Incidence of *Escherichia coli *bloodstream infection, Auckland District Health Board, New Zealand, 2005–2011.**

**Figure 2:**
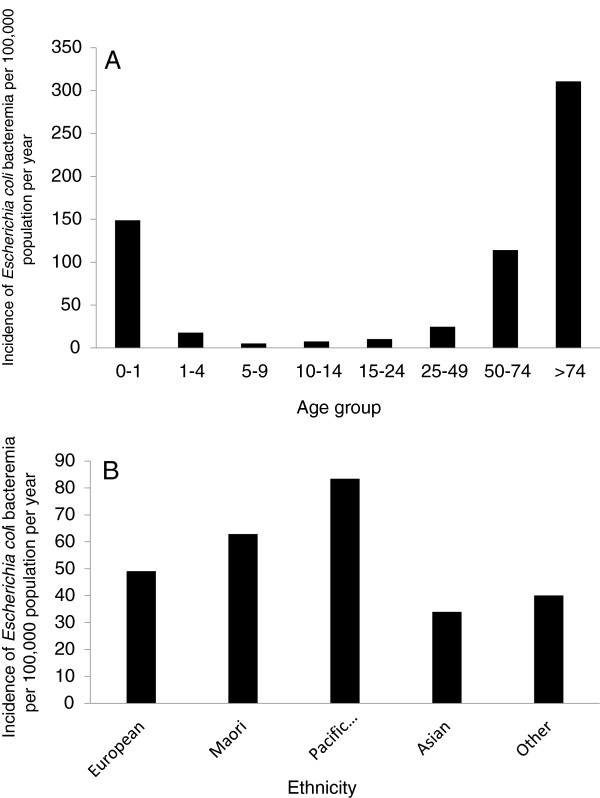
**Incidence of *Escherichia coli *bloodstream infection Auckland District Health Board, New Zealand, 2005 – 2011, stratified by age (A) and (B) ethnicity.**

**Figure 3:**
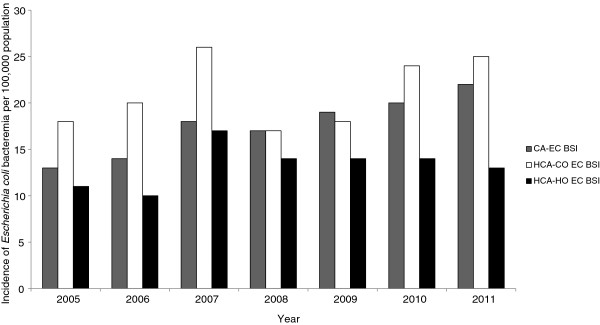
**Incidence of *Escherichia coli *bloodstream infection Auckland District Health Board, New Zealand, 2005 – 2011, stratified by place of acquisition.**

A total of 1507 patients had an episode of EC-BSI over the study period. The overall average annual incidence of EC-BSI was 52 per 100,000 population, and increased from 42 to 60 per 100,000 population over the study period (Figure 1). When stratified by age (Figure 2A), the incidence was highest in the under one and over 75 year age brackets (149 and 310 per 100,000 population respectively), and when stratified by ethnicity (Figure 2B), the incidence was highest in Māori and Pacific Peoples (83 and 62 per 100,000 population respectively). Of the 1507 episodes of EC-BSI, 510/1507 (34%) were classified as CA EC-BSI, 608/1507 (40%) were HCA-CO EC-BSI, and 389/1507 (26%) were HCA-HO EC-BSI (Figure 3). The incidences of HCA-CO and HCA-HO EC-BSI did not increase significantly over the study period; however, the incidence of CA EC-BSI increased significantly from 13 per 100,000 to 22 per 100,000 population (*P* < 0.001).

### Demographic characteristics and clinical outcomes

Full demographic data were available for 1467 patients with EC-BSI (Table 1). Of these 1467 patients, 806 (54.9%) patients were of European ethnicity, 288 (19.6%) were Pacific Peoples, 220 (15.0%) were Asian, 133 (9.1%) were Māori and 20 (1.4%) were of other ethnicities. The median age of the patients was 63 years (IQR 45 – 77 years), and was significantly lower in patients with HCA-HO infections (median 58 years; IQR 35 – 75 years) compared to patients with HCA-CO infections (median 65 years; IQR 47 – 79 years) or CA infections (median 63 years; IQR 47 – 74 years) (Table 1). Overall, 678 / 1507 (55%) patients were female; however, patients with HCA-HO infections were significantly more likely to be male than patients with CA or HCA-CO infections (Table 1). The median length of stay was 7 days (IQR 4 – 16 days), and was significantly higher in those patients with HCA-HO infections (Table 1). Overall, 216 / 1507 (14%) of patients with EC-BSI had an ICU admission, and the aggregate in-hospital mortality rate was 9%. When compared to patients with CA and HCA-CO infections, patients with HCA-HO infections were significantly more likely to have an ICU admission and were significantly more likely to die during their hospital admission (Table 1). The median duration between hospital admission and first EC-BSI in patients with HCA-HO infections was 11.4 days (IQR 7.1 – 15.2 days).

**Table 1:** **Characteristics and outcomes of *Escherichia coli *bloodstream infections, Auckland District Health Board, 2005 - 2011**

**Characteristic**	**All patients (n = 1507)**	**Community-associated (n = 510)**	**Healthcare-associated, community-onset (n = 608)**	**Healthcare-associated, hospital-onset (n = 389)**	***P *value of CA vs. HCA-CO**	***P *value of CA vs. HCA-HO**	***P *value of HCA-CO vs. HCA-HO**
Age, median, years (IQR) ^a^	63 (45 – 77)	63 (47 – 74)	65 (47–79)	58 (35–75)	0.05	0.01	< 0.001
Gender, male ^b^	678 (45)	208 (41)	255 (42)	215 (55)	0.71	< 0.001	< 0.001
Clinical outcomes							
LOS, median, days (IQR) ^a^	7 (4–16)	5 (3 – 8)	6 (4 – 10)	24 (14 – 40)	< 0.001	< 0.001	<0.001
ICU admission ^b^	216 (14)	56 (11)	50 (8)	110 (28)	0.31	< 0.001	< 0.001
In-hospital mortality ^b^	135 (9)	21 (4)	57 (9)	57 (15)	< 0.001	< 0.001	0.01
Isolate characteristics							
ESBL phenotype ^b, c^	66 (4.4)	24 (5)	30 (5)	32 (8.5)	0.89	0.04	0.04
MDR phenotype ^b^	609 (40)	180 (35)	268 (44)	161 (41)	0.003	0.07	0.43

^a^*P* value calculated using Mann Whitney *U* test.

^b^*P* value calculated using χ^2^ test.

^c^ Number of isolates tested for ESBL phenotype = 1492 (505 CA, 602 HCA-CO, 385 HCA-HO).

### Rates and trends of antimicrobial resistance in Escherichia coli bloodstream isolates

**Figure 4:**
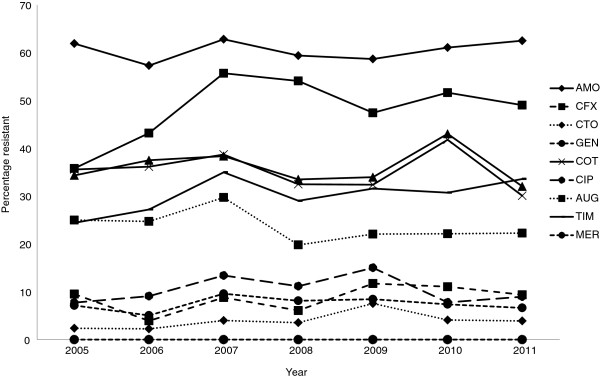
**Rates of antimicrobial resistance among *Escherichia coli *bloodstream isolates, Auckland District Health Board, New Zealand, 2005–2011.**

Antimicrobial susceptibility test results were available for 1505/1507 (99%) isolates. The overall rates of resistance were: amoxycillin, 61% (911/1505 isolates); cephalothin, 48% (724/1505); cefuroxime, 9% (132/1505); ceftriaxone, 4% (60/1505); aztreonam, 4% (57/1505); amoxycillin-clavulanate, 24% (356/1505); ticarcillin-clavulanate, 30%, 455/1505; gentamicin, 8% (113/1505); amikacin, 0.2% (3/1505); trimethoprim-sulfamethoxazole, 35% (532/1505), and ciprofloxacin, 10% (157/1505). Rates of resistance to any of the tested antimicrobials did not increase significantly over the study period (Figure 4). ESBL production was detected in 5.6% (84/1505) of isolates tested. No carbapenemase-producing *E. coli* bloodstream isolates were detected over the study period. The prevalence of ESBL-producing *E. coli* was significantly higher in HCA-HO infections when compared to HCA-CO EC-BSI infections (*P* = 0.04) or CA EC-BSI infections (*P* = 0.04). In addition, MDR - *E*. *coli* isolates were significantly less common in CA EC-BSI infections when compared to HCA-CO infections (*P* = 0.003) or HCA-HO infections (*P* = 0.07).

## Discussion

In this study, we assessed the incidence and clinical outcomes of EC-BSI in our locale, with a particular focus on possible sociodemographic variation. In addition to further highlighting the demographic differences between community and nosocomial EC-BSI, we also found notable differences across both age and ethnic groups. These findings have implications for the following reasons.

Firstly, our study provides valuable baseline data on the incidence and burden of EC-BSI in New Zealand. By utilizing a population-based approach, our data can be compared to studies in other settings that have assessed the population burden of these serious infections. We found that the overall incidence of EC-BSI in our setting (52 per 100,000 population) was higher than that reported from studies in similar developed countries. For example, Kennedy et al. reported an incidence of EC-BSI of 28 per 100,000 population per year in Canberra, Australia [12], and a recent Finnish study observed an average annual rate of 44 per 100,000 population between 2004 and 2007 [20]. The reasons for the higher observed rate of EC-BSI in our locale are unclear; however the incidence of other specific infectious diseases are also notably higher in New Zealand compared to other settings, including skin and soft tissue infections [21], campylobacteriosis [22] and meningococcal disease [23]. Similar to other studies [5,12,24], we also observed an age-related difference in the incidence of EC-BSI, with the highest incidence in the very young and elderly age groups. Unexpectedly, we found a difference in the incidence of EC-BSI across ethnicities, with rates highest in Pacific Peoples and Māori (83 and 62 per 100,000 population respectively). To our knowledge, our study represents the first to describe ethnicity-related differences in the incidence of EC-BSI, and is consistent with previous studies in our setting describing ethnic variation in other infectious diseases [25-27]. It is unclear why the incidence of EC-BSI is highest in these specific ethnic groups; however, possible reasons include barriers in accessing healthcare resulting in delayed presentation to hospital [28,29], and the higher incidence of medical co-morbidities in these populations, notably diabetes mellitus [30].

Secondly, by stratifying EC-BSI into place of acquisition, we were able to detect differences in both the incidence and demographics of community vs. healthcare-associated infections. Approximately one-third of cases occurred in patients with no prior healthcare exposure and were therefore considered to represent true CA EC-BSI. Although the incidence of HCA EC-BSI did not increase over the study period, the incidence of CA *E*. *coli* bacteremia rose significantly (*P* < 0.001). Increasing rates of CA EC-BSI have been also described in other countries [7,8]; suggested reasons for the increase in these settings include climatic factors and rising rates of antimicrobial resistance [7,8]. Similar to other studies [6,24], we found that the vast majority (74%) of cases of EC-BSI in our setting originated in the community. Interestingly however, most of these episodes occurred in patients with prior healthcare exposure, and were classified as HCA-CO EC-BSI. This finding may reflect changes in healthcare delivery, with increasingly complex medical treatments and procedures being delivered in a community setting. Recently, it has been suggested that the subgroup of HCA-CO BSI represents a distinct epidemiological entity, with overlapping demographic features of both community and nosocomial bacteremia [31]. In keeping with this suggestion, we also observed notable differences between the three acquisition categories. For example, characteristics of patients with HCA-CO EC-BSI were similar to CA EC-BSI with regards to age and gender, but had intermediate features between CA and HCA-HO EC BSI regarding clinical outcomes, such as length of stay and in-hospital mortality. In addition, we also observed differences in antimicrobial resistance phenotype between the three categories, such that the ESBL and MDR phenotypes were commoner in patients with HCA-HO infections, and the MDR phenotype was less common in CA EC-BSI infections. This finding is consistent with other studies describing healthcare exposure as a risk factor for these specific antimicrobial resistance profiles [32,33].

Finally, unlike other studies [7,8] we did not observe a significant increase in the rates of antimicrobial resistance amongst our *E*. *coli* bloodstream isolates over the study period. In general, rates of antimicrobial resistance are low in New Zealand compared to other settings. In particular, resistance rates for fluoroquinolones, aminoglycosides and third generation cephalosporins were lower than rates reported from studies in other countries [7]. Although we did not detect any carbapenemase-producing isolates in our study, these organisms have been sporadically isolated in New Zealand, specifically from travellers returning from areas with a reported high prevalence of highly resistant Enterobacteriaceae [34,35].

There were a number of limitations with our study. Most notably, our retrospective approach meant we were unable to collect information on medical co-morbidities and the source of EC-BSI. In particular, identification of the focus of infection would have allowed us to identify potentially modifiable risk factors for preventing EC-BSI. For example, a previous study in our setting found that 20% of CA EC-BSI in males was secondary to recent prostate biopsy [36]. A further limitation was our use of in-patient mortality as an outcome measure. This limited our comparison with other studies of EC-BSI that have predominantly assessed mortality rates at either seven days [12] or thirty days [2].

## Conclusions

In summary, our work provides valuable baseline data on the incidence and burden of EC-BSI in our locale. The incidence of EC-BSI was higher that that described from other developed countries, with significant demographic variation, most notably in ethnic-specific incidence rates. Future work in our setting should assess the possible reasons for these differences, in addition to identifying potentially modifiable risk factors for these serious infections.

## Abbreviations

CA: Community-associated; HCA-CO: Healthcare-associated community-onset; HCA-HO: Healthcare-associated hospital-onset; IQR: Interquartile range; LOS: Length of stay; ICU: Intensive care unit; ESBL: Extended spectrum beta-lactamase; MDR: Multidrug-resistant.

## Competing interests

The authors declare that they have no competing interests.

## Authors’ contributions

DAW conceived the study, participated in data collection and analysis, and drafted the manuscript. AL and SW participated in data collection and analysis. SR and JF provided intellectual contributions to the manuscript. All authors read and approved the final manuscript.

## Pre-publication history

The pre-publication history for this paper can be accessed here:

http://www.biomedcentral.com/1471-2334/13/385/prepub
